# Targeted disruption of pi–pi stacking in Malaysian banana lectin reduces mitogenicity while preserving antiviral activity

**DOI:** 10.1038/s41598-020-80577-7

**Published:** 2021-01-12

**Authors:** Evelyn M. Covés-Datson, Steven R. King, Maureen Legendre, Michael D. Swanson, Auroni Gupta, Sandra Claes, Jennifer L. Meagher, Arnaud Boonen, Lihong Zhang, Birte Kalveram, Zoe Raglow, Alexander N. Freiberg, Mark Prichard, Jeanne A. Stuckey, Dominique Schols, David M. Markovitz

**Affiliations:** 1 Medical Scientist Training Program, University of Michigan, Ann Arbor, MI 48109 USA; 2 Department of Microbiology and Immunology, University of Michigan, Ann Arbor, MI 48109 USA; 3 Division of Infectious Diseases, Department of Internal Medicine, University of Michigan, Ann Arbor, MI 48109 USA; 4 Graduate Program in Immunology, University of Michigan, Ann Arbor, MI 48109 USA; 5 Laboratory of Virology and Chemotherapy, Rega Institute for Medical Research, University of Leuven, 3000 Leuven, Belgium; 6 Life Sciences Institute, University of Michigan, Ann Arbor, MI 48109 USA; 7 Department of Pathology, University of Texas Medical Branch, Galveston, TX 77555 USA; 8 University of Alabama Health Services Foundation Diagnostic Virology Laboratory, University of Alabama, Birmingham, AL 35294 USA; 9 Department of Biological Chemistry, University of Michigan, Ann Arbor, MI 48109 USA; 10 Cellular and Molecular Biology Program, University of Michigan, Ann Arbor, MI 48109 USA; 11 Cancer Biology Program, University of Michigan, Ann Arbor, MI 48109 USA; 12 Present Address: Predictive and Clinical Immunogenicity, Merck and Co., Inc, Kenilworth, NJ 07033 USA

**Keywords:** Glycobiology, Antimicrobials, Virology

## Abstract

Lectins, carbohydrate-binding proteins, have been regarded as potential antiviral agents, as some can bind glycans on viral surface glycoproteins and inactivate their functions. However, clinical development of lectins has been stalled by the mitogenicity of many of these proteins, which is the ability to stimulate deleterious proliferation, especially of immune cells. We previously demonstrated that the mitogenic and antiviral activities of a lectin (banana lectin, BanLec) can be separated via a single amino acid mutation, histidine to threonine at position 84 (H84T), within the third Greek key. The resulting lectin, H84T BanLec, is virtually non-mitogenic but retains antiviral activity. Decreased mitogenicity was associated with disruption of pi–pi stacking between two aromatic amino acids. To examine whether we could provide further proof-of-principle of the ability to separate these two distinct lectin functions, we identified another lectin, Malaysian banana lectin (Malay BanLec), with similar structural features as BanLec, including pi–pi stacking, but with only 63% amino acid identity, and showed that it is both mitogenic and potently antiviral. We then engineered an F84T mutation expected to disrupt pi–pi stacking, analogous to H84T. As predicted, F84T Malay BanLec (F84T) was less mitogenic than wild type. However, F84T maintained strong antiviral activity and inhibited replication of HIV, Ebola, and other viruses. The F84T mutation disrupted pi–pi stacking without disrupting the overall lectin structure. These findings show that pi–pi stacking in the third Greek key is a conserved mitogenic motif in these two jacalin-related lectins BanLec and Malay BanLec, and further highlight the potential to rationally engineer antiviral lectins for therapeutic purposes.

## Introduction

Viral infections are a major cause of morbidity and mortality and suboptimal therapeutic options exist for many diseases of viral etiology, underscoring the urgent need for new agents to treat most viral infections. Further, unlike the case with bacteria, few broad-spectrum antiviral agents exist. A group of proteins called lectins has emerged as promising potential antivirals. Broadly defined, lectins encompass a category of proteins that bind non-enzymatically to carbohydrates, including glycoproteins found on viral and cell surfaces. Lectins recognize and bind to particular mono- or oligo-saccharides through their carbohydrate recognition domains in a specific manner that depends on the types of internal linkages between monosaccharides within the target glycan^1^. The existence of lectins was first reported by Peter Hermann Stillmark in 1888, who described a hemagglutinin isolated from the castor tree, so named for its ability to agglutinate erythrocytes, which he called ricin^2^. Since that time, lectins have been described ubiquitously, including in bacteria, viruses, plants, and animals; they are also present endogenously in humans^1,3^.

Lectins appear to play diverse roles in viral pathogenesis and the host immune response: they are capable of inhibiting viral attachment, entry, and fusion, and of activating both the innate and adaptive immune systems. For example, the transmembrane lectin Langerin, found on Langerhans cells, recognizes mannose, *N*-acetylglucosamine, and fucose monosaccharides and has been shown to bind and degrade HIV-1 in Birbeck granules^4^. The mammalian lectin mannose binding lectin (MBL), which is specific for mannose, is capable of binding to viral surfaces and activating the complement cascade, thus facilitating viral elimination^5^. Indeed, individuals with low levels of MBL may be more susceptible to infection with respiratory syncytial virus^6^. The soluble lectins surfactant proteins A and D (SP-A and SP-D), also specific for mannose, have been shown to inhibit influenza A virus activity by preventing viral attachment and entry, facilitating macrophage clearance, and activating neutrophil chemotaxis and the oxidative burst^7–9^.

While not all lectins are mitogenic, many lectins are mitogenic and the potential of these proteins as promising prophylactic and therapeutic antiviral agents has been limited by their mitogenicity. The mitogenic effects of certain lectins were first reported in 1960 by Nowell, who described the ability of the plant lectin phytohemagglutinin (PHA) to induce the growth and division of lymphocytes^10^. Lectins exert their mitogenic activity through multiple mechanisms, including binding to T cell receptors (TCRs) and inducing second messenger-mediated stimulation and production of pro-proliferation cytokines^11,12^. Indeed, this property of lectins has been utilized by the scientific community to study mechanisms of immune activation, immunodeficiencies, and malignancies, and in other applications such as growing retroviruses. However, the mitogenicity of many lectins presents a significant barrier to their more widespread clinical use as antivirals. For example, the prokaryote-derived lectin cyanovirin-N (CVN), which binds to α(1,2) mannose and has anti-HIV activity, was shown to have marked stimulatory effects on treated cells. This stimulation could paradoxically increase susceptibility to HIV through activation of chemokines and T cells, upregulation of activation markers, and subsequent recruitment of susceptible immune cells to sites of infection^13^. On a broader level, if lectins are to be used as antivirals, one of the most important potential routes of administration is the mucosal surface. Nonspecific, uncontrolled inflammation of the mucosa would likely increase viral transmission by facilitating breaks in the epithelium; thus, if the mitogenic and inflammatory effects of lectins cannot be mitigated, they could paradoxically increase the viral transmission they are intended to prevent.

Banana lectin (BanLec), a high-mannose-specific lectin isolated from the fruit of bananas, and its molecularly engineered H84T variant (see below) have been shown to have antiviral activity through the inhibition of cellular attachment, viral entry, and endosomal fusion of viruses including HIV^14,15^, HCV^15^, influenza^16^, and Ebola^17^. However, the form of BanLec that is directly purified from the fruit also has significant mitogenic activity, and has been shown to induce T cell proliferation and cytokine production^15,18^, as well as cause local injection site reactions, piloerection, and weight loss in mice^16^, thus limiting its potential therapeutic use. In 2015, we demonstrated that through rational molecular engineering, it is possible to significantly decrease lectin mitogenicity while preserving antiviral activity^15^. This was, to our knowledge, the first example of the ability to separate these two distinct functions of a lectin through molecular engineering. The decrease in mitogenicity was achieved through a specific amino acid substitution in which a histidine at position 84 was changed to a threonine (H84T), which, through disruption of pi–pi stacking in the third Greek key of BanLec, appears to diminish the capacity of H84T BanLec (H84T) to interact with multiple glycan molecules, which is required for mitogenicity. However, importantly, H84T retains the potent antiviral activity of wild-type (WT) BanLec with similar carbohydrate-binding specificity, though with slightly reduced binding avidity. H84T was one of only two mutants that exhibited reduced mitogenicity with preserved antiviral activity; all other substitutions abolished both mitogenicity and antiviral activity, presumably due to decreased carbohydrate-binding activity^15^. Although disruption of pi–pi stacking in H84T is associated with its decreased mitogenicity, it remained to be determined whether disruption of this interaction in another mitogenic antiviral lectin could reduce mitogenic activity without sacrificing antiviral potential.

In this work, we thus sought to understand whether we could recapitulate in another lectin the ability to separate two distinct lectin functions via targeted molecular engineering. That is, we asked whether disruption of pi–pi stacking in a structurally related but distinct (only 63% identical at the amino acid level) jacalin-related lectin, Malaysian banana lectin (Malay BanLec), would reduce mitogenicity of the engineered lectin without abolishing antiviral activity. We demonstrate that a single amino acid substitution, F84T, akin to the H84T mutation and predicted to remove the pi–pi stacking interaction, does indeed decrease mitogenicity while preserving antiviral activity. F84T still maintains some mitogenic activity, but it stimulates peripheral blood lymphocytes (PBLs) to proliferate to a lesser degree than does WT Malay BanLec. In addition, F84T treatment leads to the production of fewer cytokines and chemokines from peripheral blood mononuclear cells (PBMCs) and may activate fewer CD4 + PBMCs than does WT Malay BanLec treatment. Antiviral activity against HIV is maintained, and F84T has robust antiviral activity against human cytomegalovirus, and Ebola, varicella-zoster, and Lassa fever viruses. These results serve as a further proof-of-principle that pi–pi stacking in the third Greek key of a jacalin-related lectin is a determinant of mitogenicity that may be disrupted to reduce mitogenic activity without removing antiviral activity. This discovery may signal new therapeutic potential for lectins, confirming that molecular engineering may allow for more widespread and targeted use of lectins through improvements in their clinical safety profiles.

## Results

### Malay BanLec is structurally similar to BanLec

**Figure 1:**
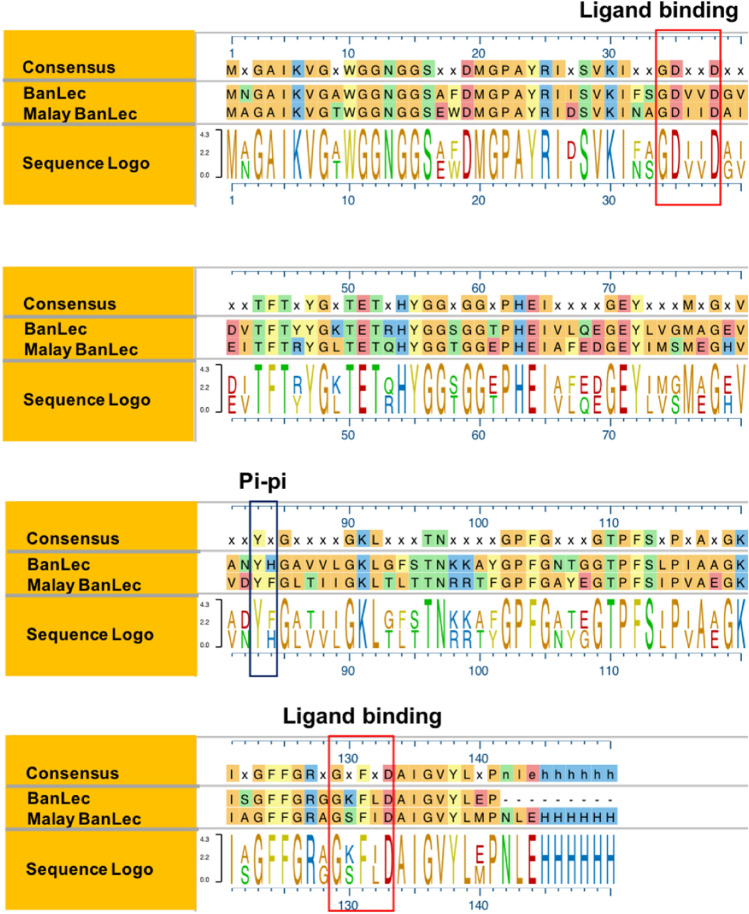
Malay BanLec is structurally similar to BanLec. Amino acid sequence alignment of BanLec and Malay BanLec (middle rows) from amino acids 1 (top left) to 141 (bottom right). The 6 × His-tag (LEHHHHHH) is indicated at the end of the Malay BanLec sequence. The amino acids predicted to undergo pi–pi stacking (“pi–pi”) and the predicted ligand binding loops of the carbohydrate binding sites (“ligand binding”) in Malay BanLec are outlined in black and red, respectively, aligned with those known sites in BanLec. Sites of amino acid identity (“consensus”) are indicated in the top row in the colored boxes; “x” indicates that identity is not shared at a particular site. The bottom row (“sequence logo”) depicts the sequence of both lectins, with amino acids from both sequences represented if there is not identity at a particular site. Malay BanLec is 63% identical to BanLec at the amino acid level. The figure was generated using Microsoft PowerPoint 2016 (https://www.microsoft.com/en-us/microsoft-365/powerpoint) and Adobe Illustrator, version 22.1.0 (https://www.adobe.com/products/illustrator.html).

Our previous work demonstrated that a single amino acid substitution (H84T) in an antiviral, mitogenic banana lectin (BanLec) led to a significant reduction in its mitogenicity^15^. Loss of the pi–pi stacking interaction between tyrosine 83 and histidine 84 in the ligand recognition loop of the third Greek key of the lectin was associated with this decrease; this loop is located between the two carbohydrate binding sites (CBS) of the lectin and had been thought to perhaps refine binding specificity to complex glycans^19^ and to control binding to multiple glycans, which promotes mitogenicity^15^. We aimed to determine whether disruption of pi–pi stacking in a structurally related but distinct lectin could similarly decrease the mitogenic activity of another antiviral lectin. To do so, we first sought to identify another lectin predicted to have two CBS separated by a ligand recognition loop containing two aromatic amino acids capable of pi–pi stacking. A BLAST protein sequence alignment search revealed a candidate lectin from *Musa acuminata* ssp. *malaccensis*, which we term Malayasian banana lectin (Malay BanLec) (Fig. 1). Malay BanLec was predicted to have the desired structural features, including two CBS ligand binding loops characterized by the GXXXD sequence^20^. Between the two CBS, as in wild-type (WT) BanLec, Malay BanLec has a tyrosine at position 83 (Y83), but instead of a histidine at position 84 as in WT BanLec, Malay BanLec has a phenylalanine (F84). We would expect Y83 and F84 in Malay BanLec to interact via pi–pi stacking. Thus, although Malay BanLec is quite a different lectin than BanLec with only 63% amino acid identity (differing at 52 of 141 amino acids), it is predicted to have important structural features in common with BanLec that make it a suitable candidate to engineer in a similar fashion, with a view to reducing mitogenicity while retaining antiviral activity.

### Malay BanLec has both mitogenic and antiviral activity

**Figure 2:**
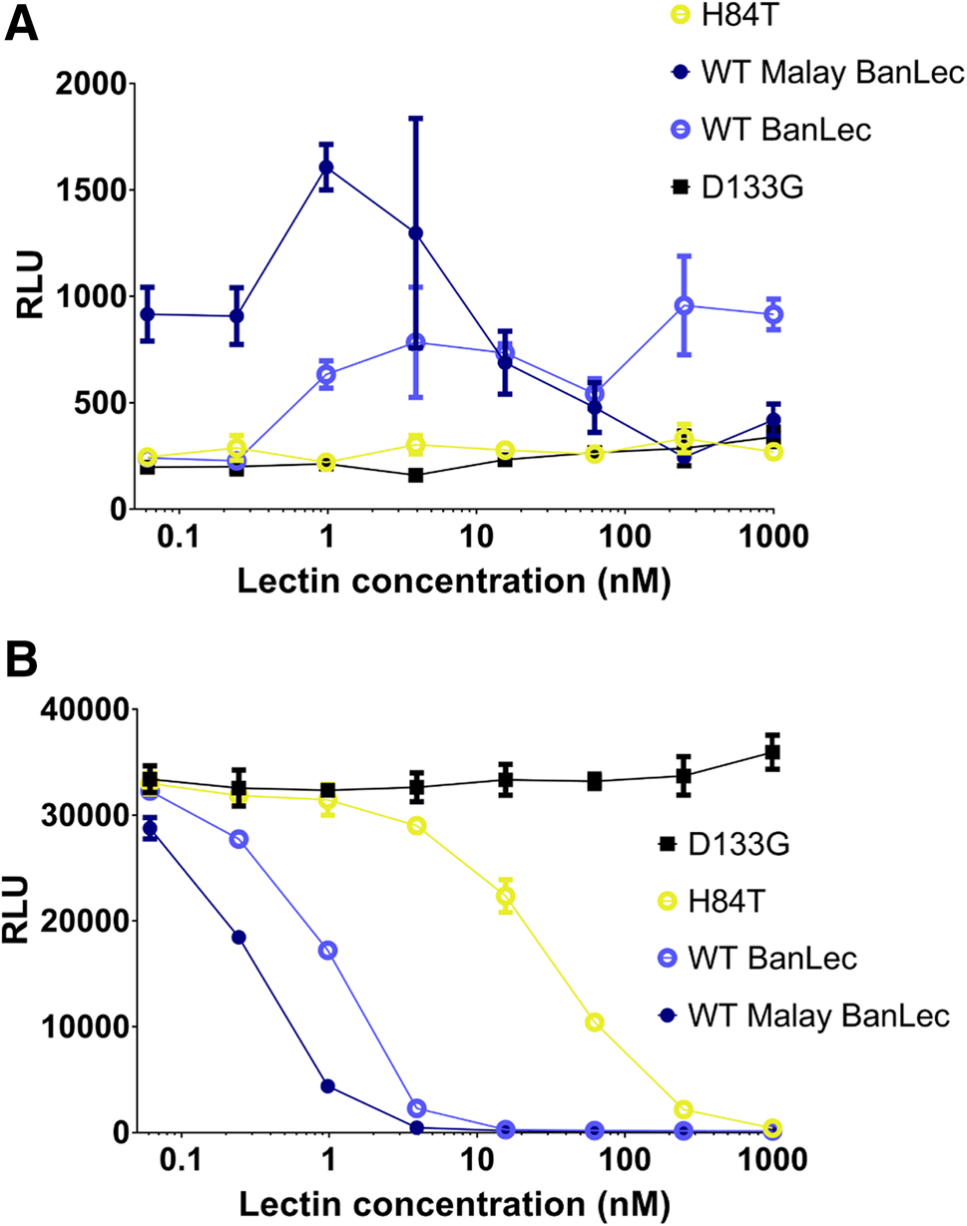
Malay BanLec has both mitogenic and antiviral activity. (**a**) BrdU incorporation into peripheral blood lymphocytes (PBLs). PBLs from a healthy human donor were treated with a range of concentrations of WT, H84T, or D133G BanLec, or Malay BanLec for 3 d and then BrdU incorporation over 18 h was measured by chemiluminescent ELISA. *RLU* relative light units. (**b**) TZM-bl cells were pre-treated with the same lectins as in A and exposed to HIV BaL for 2 days. To assess levels of infection, luciferase activity was measured. Data in A and B are representative of one independent experiment each. Error bars represent the SEM of triplicate values. The figure was generated using GraphPad Prism 7 (https://www.graphpad.com/scientific-software/prism/) and Adobe Illustrator, version 22.1.0 (https://www.adobe.com/products/illustrator.html).

First, we assessed whether Malay BanLec was mitogenic and antiviral as we expected from its predicted structural similarity with BanLec. We cloned the codon-optimized sequence of Malay BanLec containing a 6 × His-tag with the sequence LEHHHHHH into the pET9a expression vector suitable for protein expression in *E. coli*. To assess the mitogenicity of Malay BanLec, we incubated peripheral blood lymphocytes (PBLs) isolated from a healthy donor with a range of concentrations of WT, H84T, or D133G (non-carbohydrate-binding^15^) BanLec or with Malay BanLec for three days and then monitored incorporation of bromodeoxyuridine (BrdU) over 18 h by ELISA (Fig. 2A). As expected, treatment with D133G, which is neither antiviral nor mitogenic, resulted in minimal incorporation of BrdU. H84T treatment, which we have previously shown to be non-mitogenic, also resulted in minimal BrdU incorporation. In contrast, consistent with its known mitogenicity, WT BanLec caused PBLs to incorporate BrdU in a dose-dependent fashion, with increasing amounts of lectin resulting in increased incorporation of BrdU. Malay BanLec treatment resulted in greater BrdU incorporation than did WT BanLec treatment at concentrations up to 10 nM, but less incorporation above that concentration, which may be due to cell death triggered by excessive mitogenic stimulation. These results indicate that Malay BanLec is indeed mitogenic, and highly so as it is even more stimulatory than WT BanLec. To assess whether Malay BanLec has antiviral activity, we pretreated TZM-bl cells, which express a luciferase reporter for HIV infection, with varying concentrations of WT, H84T, or D133G BanLec, or Malay BanLec, and exposed them to HIV for 2 days, while maintaining the lectins in the media (Fig. 2B). Cells treated with D133G at even the highest concentration showed robust luciferase activity, indicating that D133G did not inhibit HIV infection. In contrast, treatment with all other lectins led to a dose-dependent decrease in luciferase activity with increasing concentrations of lectin. H84T exhibited the least potent inhibition of viral infection, whereas Malay BanLec exhibited the most. Therefore, Malay BanLec, like WT and H84T BanLec, does possess antiviral activity and perhaps has more potent activity against certain strains of HIV than does either WT or H84T BanLec. It should be noted that relative potency in the TZM-bl assay does not necessarily correlate with assays in primary cells. Specifically, while H84T shows less activity against HIV than does WT BanLec in TZM-bl cells, it showed at least equivalent activity against a wide range of HIV isolates when tested in primary cells^15^. (Also see below and Table 3 in this paper.)

Our previous work showed that it is not merely a matter of mutating the aromatic amino acid at position 84 in BanLec that allows for successful uncoupling of mitogenicity and antiviral activity, but also that the substituted amino acid is a threonine^15^. Therefore, we next set out to generate a similar mutation in Malay BanLec as was done with WT BanLec. We designed primers to introduce the F84T mutation by changing the F84 codon to a threonine codon with the fewest number of nucleotide changes possible. We successfully generated the F84T mutation as confirmed by sequence analysis and were able to produce a recombinant protein in *E. coli* of the predicted size as assessed by Western blotting.

### F84T Malay BanLec is less mitogenic than its WT counterpart

**Figure 3:**
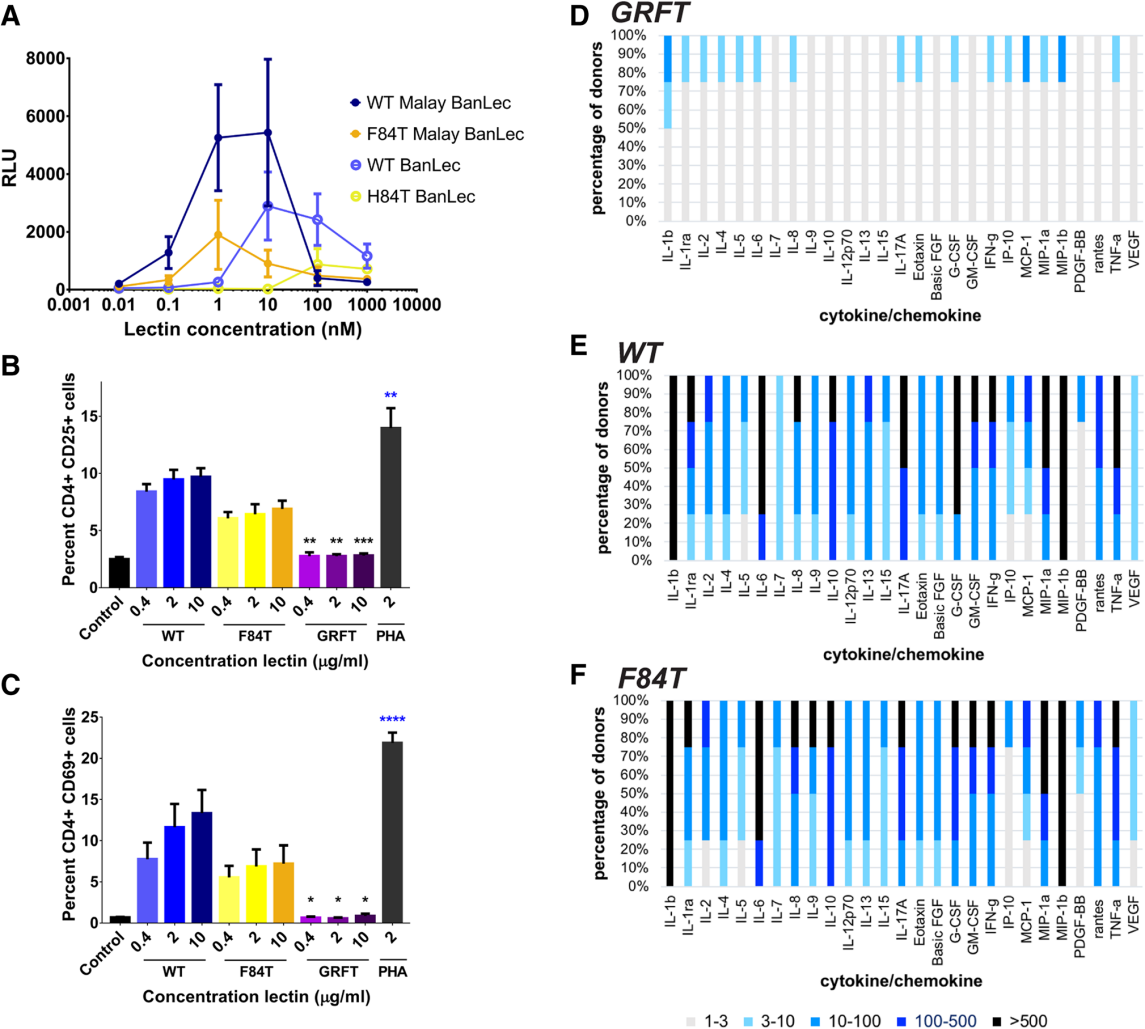
F84T Malay BanLec is less mitogenic than is WT Malay BanLec. (**a**) BrdU incorporation into PBLs. PBLs from healthy human donors were treated with a range of concentrations of WT or F84T Malay BanLec, or WT or H84T BanLec for 3 d and then BrdU incorporation over 18 h was measured by chemiluminescent ELISA. Data are representative of two independent experiments using cells from two donors, with data from one donor shown. Error bars represent the SEM of triplicate values. (**b**,**c**) Activation markers in lectin-treated peripheral blood mononuclear cells (PBMCs). PBMCs from three healthy human donors were treated with a range of concentrations of WT Malay BanLec (WT) or F84T Malay BanLec, griffithsin (GRFT), or PHA and the percentage of CD4 + CD25 + (B) or CD4 + CD69 + (C) cells assessed by flow cytometry. GRFT, known to be virtually non-mitogenic, serves as the negative control, whereas PHA serves as the positive control. Bars represent the average values from the three donors and error bars the SEM. Black asterisks indicate statistically significant differences between the means in the GRFT group and the corresponding means in the WT group. Blue asterisks indicate statistically significant differences between the mean in the PHA group and the mean in the control group. *P < 0.05, **P < 0.01, ****P < 0.0001. (**d**–**f**) Cytokine/chemokine secretion in lectin-treated PBMCs. PBMCs from multiple healthy donors were treated with GRFT (**d**), WT (**e**), or F84T Malay BanLec (**f**) and supernatants collected for analysis of expression of various cytokines and chemokines by BioPlex assay. Results are expressed as the percentage of PBMC donors whose stimulated PBMCs express the indicated fold increase values of cytokine/chemokine concentrations with respect to untreated PBMCs. The figure was generated using GraphPad Prism 7 (https://www.graphpad.com/scientific-software/prism/) and Adobe Illustrator, version 22.1.0 (https://www.adobe.com/products/illustrator.html).

To compare the mitogenicity of WT versus F84T Malay BanLec, we exposed PBLs to WT or F84T Malay BanLec for 3 days and then measured incorporation of BrdU over 18 h by ELISA (Fig. 3A). For comparison, PBLs were exposed to WT or H84T BanLec in parallel. BanLec is a known potent T cell mitogen^21^. In two different PBL donors, as expected, H84T-treated PBLs incorporated less BrdU than those that were treated with WT BanLec. F84T-treated cells also incorporated less BrdU than WT Malay BanLec-treated cells, indicating that F84T is less mitogenic than WT Malay BanLec. However, though less mitogenic than its parent lectin, F84T retained some of the mitogenicity of WT Malay BanLec and caused PBLs to proliferate more than either H84T or WT BanLec.

Given that F84T treatment induced fewer PBLs to proliferate than did WT Malay BanLec treatment, we reasoned that CD4 + cells would become less activated in response to F84T as compared to WT Malay BanLec treatment. We therefore treated peripheral blood mononuclear cells (PBMCs) from three donors with WT or F84T Malay BanLec for 72 h and examined expression of activation markers (CD25 and CD69) on CD4 + PBMCs by flow cytometry (Fig. 3B,C). PBMCs were also treated with griffithsin (GRFT), a non-mitogenic antiviral lectin^22,23^, as a negative control and PHA, which is highly mitogenic, as a positive control. CD25 is the alpha chain of the IL-2 receptor and considered a mid-stage marker of T cell activation; CD69 is considered an early stage marker of T cell activation. As expected, PHA induced high expression of both CD25 (Fig. 3B) and CD69 (Fig. 3C), whereas GRFT did not. Although the differences were not statistically significant, F84T-treated CD4 + PBMCs tended to express less CD25 (Fig. 3B) and less CD69 (Fig. 3C) than those treated with WT Malay BanLec, suggesting that they were less activated and supporting the finding that F84T induces less proliferation of PBLs than does WT Malay BanLec. There appeared to be a dose effect trend, with higher concentrations of either lectin inducing higher expression of activation markers, more so with CD69 than CD25. Variation in human donor PBMC responses likely accounts for the lack of statistically significant data, as examination of the individual responses reveals clear differences between the F84T- and WT Malay BanLec-treated cells (Table 1).

**Table 1:** Expression of cellular activation markers by F84T- versus WT-treated CD4 + PBMCs. The percent of cells positive for each marker is shown.

		Donor 1		Donor 2		Donor 3	
		CD4 + CD25 +	CD4 + CD69 +	CD4 + CD25 +	CD4 + CD69 +	CD4 + CD25 +	CD4 + CD69 +
Control		2.69	0.60	2.14	0.67	2.69	0.78
WT	0.4 μg/ml	8.02	5.10	7.50	6.42	9.68	11.70
	2 μg/ml	9.28	7.96	8.09	9.74	11.00	17.20
	10 μg/ml	10.40	7.73	8.18	15.60	10.50	16.70
F84T	0.4 μg/ml	5.31	3.63	5.73	4.75	7.12	8.26
	2 μg/ml	5.27	3.75	5.83	6.06	8.13	10.80
	10 μg/ml	5.87	3.83	6.45	6.40	8.29	11.40
GRFT	0.4 μg/ml	3.44	0.36	2.34	0.78	2.51	0.77
	2 μg/ml	2.94	0.32	2.41	0.72	2.94	0.62
	10 μg/ml	3.18	0.46	2.54	1.41	2.73	0.68
PHA	2 μg/ml	17.40	23.80	11.70	19.50	12.80	22.30

In order to further refine our understanding of the mitogenic differences between WT and F84T Malay BanLec, we next treated PBMCs from multiple healthy donors with WT or F84T Malay BanLec, or with GRFT for 72 h (Fig. 3D–F). Supernatants from all lectin-treated PBMCs were collected and analyzed for the expression of a panel of cytokines and chemokines by Bio-Plex assay. Cytokine and chemokine signals secreted by lymphocytes in response to mitogenic stimuli cause them to proliferate^12^. GRFT stimulated only minimal expression of cytokines and chemokines. Consistent with the two assays above, F84T-treated PBMCs induced the expression of cytokines and chemokines, but overall, greater cytokine and chemokine expression was seen in WT Malay BanLec-treated cells. F84T and WT Malay BanLec stimulated similar percentages of PBMCs to secrete IL-1β, IL-4, IL-5, IL-6, IL-10, IL-12p70, IL-15, eotaxin, basic FGF, GM-CSF, IFN-γ, MCP-1, MIP-1α, MIP-1β at similar levels. F84T treatment induced somewhat higher expression of IL-7, IL-8, IL-9, PDGF-BB from a greater percentage of PBMCs as compared to WT treatment. However, a greater percentage of WT-treated PBMCs expressed higher levels of IL-1RA, IL-2, IL-13, IL-17A, G-CSF, IP-10, RANTES, TNF-α, and VEGF as compared to those treated with F84T. IL-2 is an important stimulus for progression of T cells to S phase^12^, so it is notable that F84T stimulates less expression of this cytokine from PBMCs than does WT. Additionally, IL-1RA, IL-17, TNF-α, and VEGF are all key inflammatory cytokines that have been targeted clinically.

Taken together, these studies indicate that F84T induces less proliferation of PBLs, less expression of key cytokines/chemokines from PBMCs, and perhaps less activation of CD4 + PBMCs than WT Malay BanLec, indicating that F84T is less mitogenic than WT Malay BanLec. It still does, however, possess rather significant mitogenic properties, perhaps due to other structural determinants of mitogenicity that have yet to be elucidated (see “Discussion”). In addition, we must note that we may possibly be underestimating the reduction in mitogenicity of F84T as compared to WT Malay BanLec. For unclear reasons the removal of endotoxin from Malay BanLec, and especially F84T, is difficult and hence incomplete, which might possibly contribute to mitogenicity as endotoxin is a B cell mitogen. Previous work with BanLec and H84T BanLec, originally performed when we did not routinely remove endotoxin from the preparations^15^, suggests that these assays are not sensitive to certain levels of endotoxin, since H84T BanLec preparations from which endotoxin has and has not been removed are both not mitogenic to PBLs. However, the levels of endotoxin in the preparations of F84T are much higher. That the preparation of F84T contained markedly more endotoxin than that of WT Malay BanLec (when endotoxin removal was attempted, > 10 versus 2.07 endotoxin units/mg, respectively) and was still less stimulatory to PBLs indicates that the F84T mutation did, as expected, decrease mitogenicity of the lectin and moreover suggests that the decrease in mitogenic activity in F84T may perhaps be underestimated.

### F84T Malay BanLec retains robust antiviral activity

**Figure 4:**
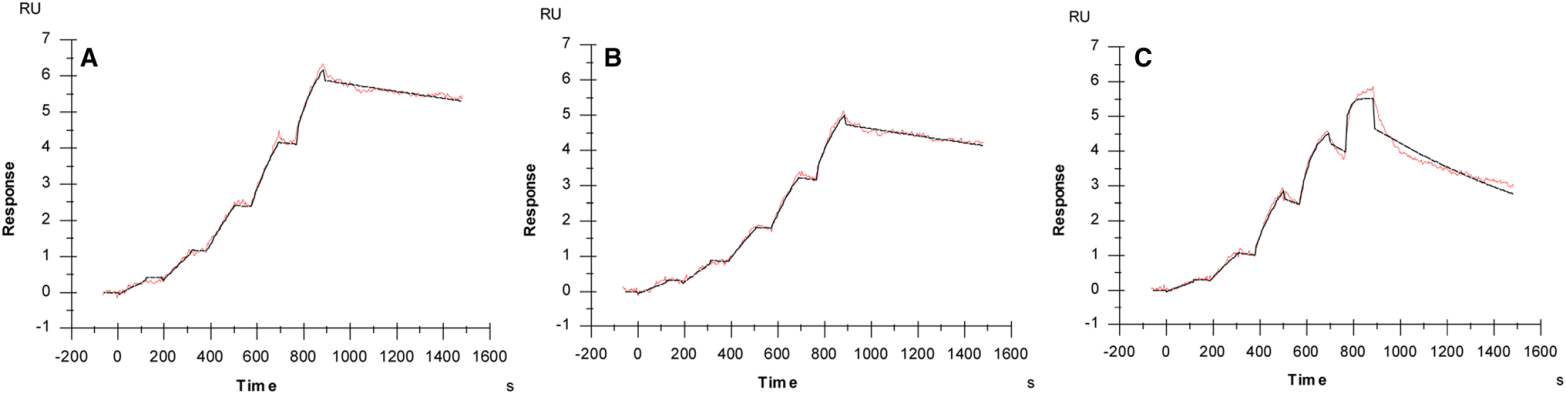
F84T Malay BanLec binds to HIV gp140 as determined by surface plasmon resonance. Single cycle kinetics of WT Malay BanLec (**a**), F84T Malay BanLec (**b**) and GRFT (**c**) binding to gp140. The concentrations of the injected lectins ranged from 0.63 to 10 nM for WT and F84T Malay BanLec and from 0.12 to 10 nM for GRFT. The red lines indicate the actual response measured during the experiment; the black lines indicate the 1:1 Langmuir model fitting. WT and F84T Malay BanLec bind 4 and 8 times slower, respectively, to the gp140 trimer than does GRFT and dissociate 5 and 3 times slower, respectively, than does GRFT. The binding avidities, *K*_D_, of WT Malay BanLec, F84T, and GRFT are 1.22 ± 0.62, 3.68 ± 0.41, 1.37 ± 0.35 (10^–10^) (M), respectively. The graphs depict one of the three replicate experiments performed. The figure was generated using Biacore T200 Evaluation Software 3.1 (https://www.cytivalifesciences.com/en/us/shop/protein-analysis/spr-label-free-analysis/systems/biacore-t200-p-05644) and Adobe Illustrator, version 22.1.0 (https://www.adobe.com/products/illustrator.html).

Before assessing whether F84T maintained antiviral activity against HIV, we first sought to assess whether F84T could bind HIV, as certain mutations (like D133G) in lectins abolish carbohydrate-binding activity^15^, which in turn disrupts antiviral activity. HIV is highly *N*-glycosylated and BanLec, as a high-mannose-binding lectin, binds to HIV and inhibits attachment of the virus to host cells^14^. With its structural similarity to BanLec, Malay BanLec is predicted to also bind to HIV, which is supported by its inhibition of the virus (Fig. 2B). To assess binding of WT versus F84T to HIV, we conducted surface plasmon resonance analysis to determine the binding times to, and binding avidities for, the gp140 trimer, which is made up of uncleaved ectodomains of the gp160 HIV envelope glycoprotein trimer (Fig. 4). The results show that F84T and WT Malay BanLec, which like BanLec exist as dimers with two CBS per monomer (see Fig. 6 below and discussion thereof), bind with high avidity to the gp140 trimer. This was expected as these lectins bind to high-mannose structures that are highly expressed on the trimer. Even after optimization of the assay, the WT and F84T Malay BanLec showed linear association curves and the evaluation software also indicated that mass transport limitation was present; the mass transport coefficient was taken into account in the fitting model. It is clear that WT and F84T Malay BanLec bind 4 and 8 times slower, respectively, to the gp140 trimer than does GRFT and dissociate 5 and 3 times slower, respectively, than does GRFT. Even though their kinetic properties differ, WT Malay BanLec and GRFT obtain quite similar binding avidities (K_D_) and F84T Malay BanLec has a slightly higher K_D_ (Table 2).

**Table 2:** Average and standard deviation of kinetics obtained from three replicate SPR experiments measuring the binding response between BG505 SOSIP.664 gp140 trimers and lectins (WT Malay BanLec, F84T Malay BanLec, and GRFT).

gp140 chip			
Sample	*k*_a_ (10^6^)(1/M.s)	*k*_d_ (10^–4^)(1/s)	*K*_D_ (10^–10^)(M)
WT	1.33 ± 0.36	1.40 ± 0.25	1.22 ± 0.62
F84T	0.72 ± 0.03	2.66 ± 0.29	3.68 ± 0.41
GRFT	5.56 ± 0.80	7.40 ± 1.09	1.37 ± 0.35

*k*_a_ association rate constant, *k*_d_ dissociation rate constant, *K*_D_ binding avidity.

**Figure 5:**
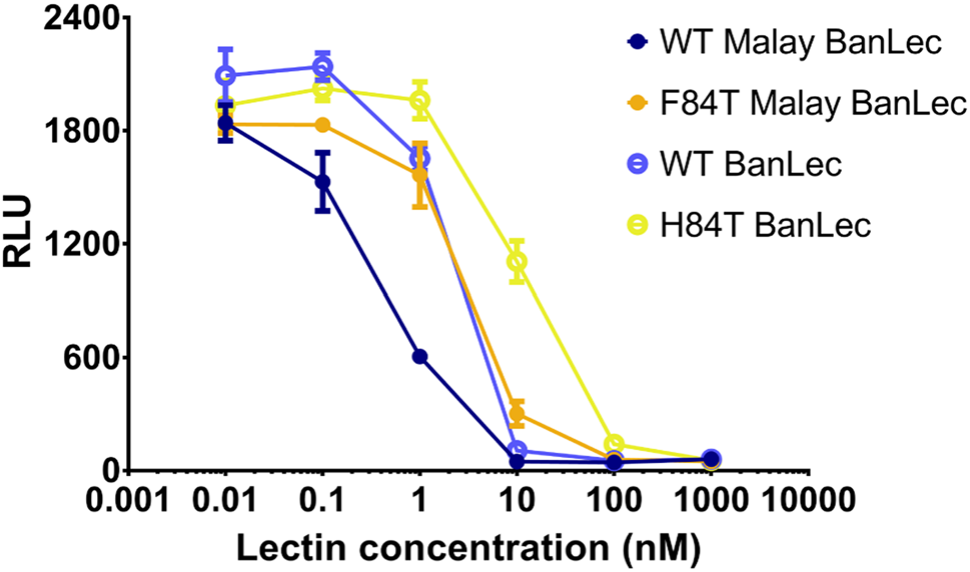
F84T retains antiviral activity against HIV. TZM-bl cells were pre-treated with WT or F84T Malay BanLec, or WT or H84T BanLec and exposed to HIV BaL for 2 d. To assess levels of infection, luciferase activity was measured. Data are representative of two independent experiments. Error bars represent the SEM. The figure was generated using GraphPad Prism 7 (https://www.graphpad.com/scientific-software/prism/) and Adobe Illustrator, version 22.1.0 (https://www.adobe.com/products/illustrator.html).

Having confirmed that F84T retains the ability to bind to the HIV glycoprotein, we next assessed whether it maintained anti-HIV activity. To do so, we pretreated TZM-bl cells with F84T or WT Malay BanLec, or with H84T or WT BanLec, exposed them to HIV for 2 days in the continued presence of lectins, and measured luciferase activity as a readout for HIV infection. As shown in Fig. 5, WT BanLec had more potent antiviral activity than did H84T BanLec, and WT Malay BanLec exhibited the most potent anti-HIV activity. F84T robustly reduced luciferase activity, indicating that it retained the ability to inhibit HIV infection. The potency of F84T in this assay was similar to that of WT BanLec. From this and the above study, it is clear that F84T binds to the HIV glycoprotein and maintains anti-HIV activity.

To further characterize the antiviral activity of F84T, we tested its ability to inhibit various strains of HIV as compared to that of WT Malay BanLec via two approaches (Table 3). Inhibitors of the HIV co-receptors CXCR4 and CCR5, AMD3100 and maraviroc, respectively, were included as controls. First, CD4 + MT-4 cells, which are CXCR4-positive but CCR5-negative, were pre-treated with a range of concentrations of lectin (F84T or WT Malay BanLec) or AMD3100 and then incubated with three different X4 strains of HIV-1 and HIV-2 for 5 days. CPE was measured to calculate 50% inhibitory concentration (IC_50_) values. Secondly, PBMCs were pre-treated as above with lectin, AMD3100, or maraviroc before exposure to CXCR4-, CCR5-, or dual-tropic HIV-1 strains for 10 days. Supernatants containing virus were collected and viral production measured by HIV-1 p24 capsid antigen ELISA to calculate IC_50_ values. F84T inhibited all strains of HIV-1 (with all tropisms) and HIV-2 tested in these assays in a low nanomolar to low micromolar range. F84T was found to have a lower IC_50_ value than WT Malay BanLec against two of three strains tested in MT-4 cells and a higher IC_50_ value than WT Malay BanLec against two of the three strains tested in PBMCs, so overall it appears that the antiviral activity of F84T against HIV is not only preserved but also not diminished.

**Table 3:** Inhibition of multiple strains of HIV by F84T versus WT Malay BanLec. CC_50_, 50% cytotoxic concentration.

MT-4	IC_50_ (ng/ml)			CC_50_ (μg/ml)
	HIV-1 NL4.3	HIV-1 HE	HIV-2 ROD	
WT	24.3	18.5	23.6	1.7
F84T	34.6	6.3	18.5	> 20
AMD3100	15.3	11.8	7.4	> 10

PBMC	IC_50_ (ng/ml)			CC_50_ (μg/ml)
	HIV-1 NL4.3 (X4)	HIV-1 HE (dual)	HIV-1 BaL (R5)	
WT	8.3	36.5	143.8	> 1
F84T	29.6	66.5	122.8	> 1
AMD3100	17.1	> 100	> 100	> 1
Maraviroc	> 100	> 100	4.9	> 1

As H84T is known to have broad-spectrum antiviral activity, we next sought to characterize whether F84T could inhibit additional viruses that are associated with human disease and are known to express mannose-containing *N*-glycans on their surfaces (Table 4). F84T had moderate to high activity against varicella-zoster, human cytomegalovirus, Ebola, and Lassa fever viruses, indicating that like H84T it has broad-spectrum antiviral activity.

**Table 4:** Inhibition of multiple viruses by F84T Malay BanLec.

Virus	Strain	Cell line	EC50	CC50
Varicella-zoster	Ellen	HFF	0.021 μM^a^	0.4 μM^a^
	15–6-001 (acyclovir-resistant isolate)	HFF	0.016 μM^a^	0.4 μM^a^
Human cytomegalovirus	AD169	HFF	< 0.05 μM^b^	2.93 μM^b^
	GDGrK17 (ganciclovir-resistant isolate)	HFF	0.08 μM^c^	7.21 μM^b^
Ebola	Zaire	Vero	< 0.1 μg/ml^d^	4.9 μg/ml^a^
Lassa fever	Josiah	Vero	0.3 μg/ml^d^	4.95 μg/ml^a^

Assay used to determination the 50% effective concentration (EC_50_) or 50% cytotoxic concentration (CC_50_).

^a^Neutral red.

^b^CellTiter-Glo.

^c^qPCR for viral DNA.

^d^Crystal violent plaque reduction.

### The F84T mutation disrupts pi–pi stacking without significant structural changes

**Figure 6:**
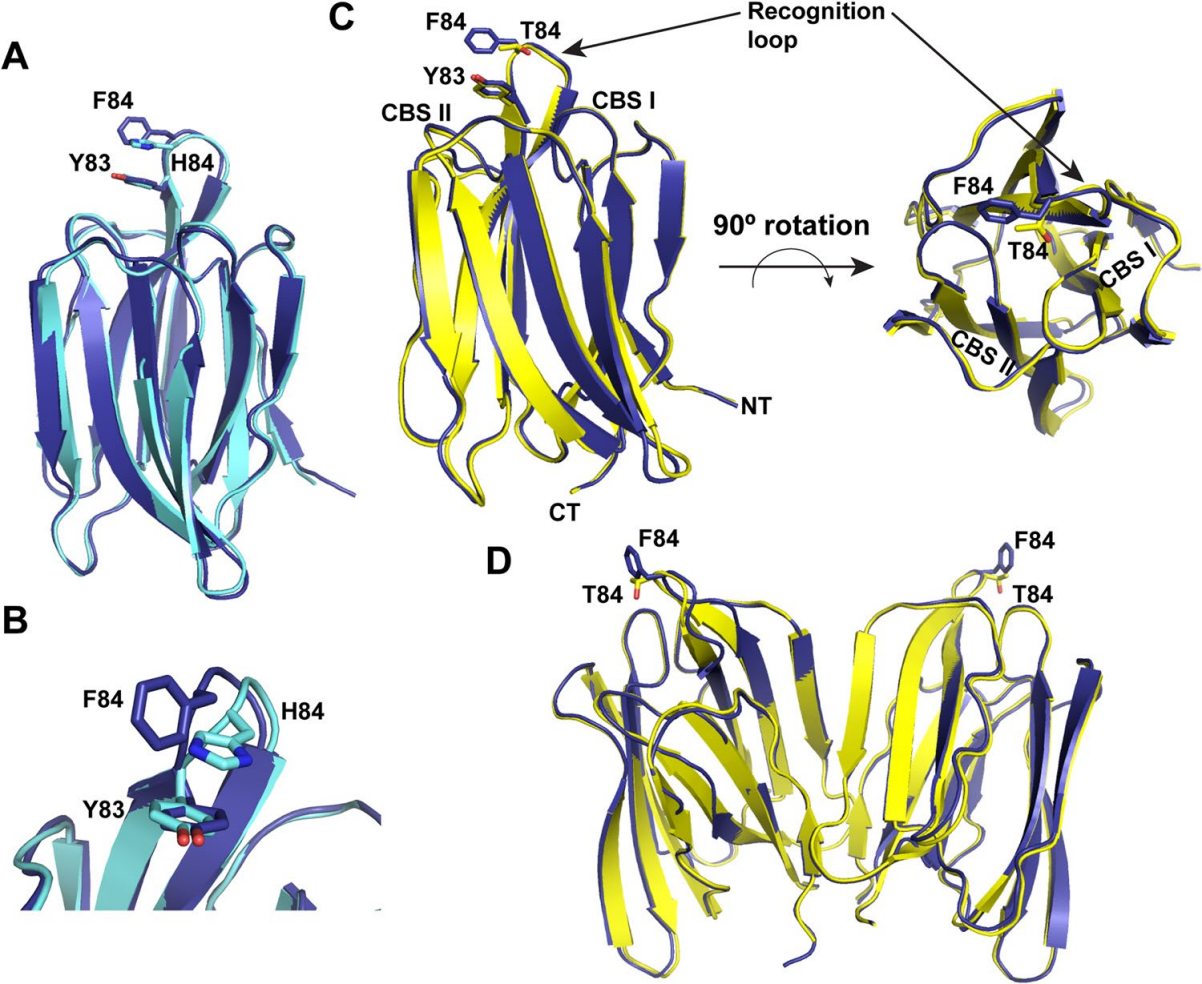
The F84T mutation interrupts pi–pi stacking between Y83 and F84 without altering the overall structure of Malay BanLec. (**a**,**b**) Crystal structures comparing WT BanLec (cyan) to WT Malay BanLec (navy). H84 in BanLec and F84 in Malay BanLec are indicated. Y83 exists in both structures. In (**a**) the alignment of the monomers is depicted, and in (**b**) a close-up of the ligand binding loops showing the pi–pi stacking interactions between H84 and Y83 and F84 and Y83 in WT BanLec and Malay BanLec, respectively. (**c**,**d**) Crystal structures comparing WT Malay BanLec (navy) to F84T Malay BanLec (yellow). In (**c**) two views of the alignment of the monomers are depicted. F84 in WT Malay BanLec and T84 in F84T Malay BanLec are indicated, along with the Y83 in common. CBS I and II are also shown, with the ligand binding loop containing the residues of interest in between them. *NT* N-terminus, *CT* C-terminus. In (**d**) the alignment of the dimers is depicted. The figure was generated using PyMol v2.3 (https://pymol.org/2/) and Adobe Illustrator, version 22.1.0 (https://www.adobe.com/products/illustrator.html).

Given that the antiviral activity of F84T was maintained, we predicted that the F84T mutation did not significantly alter the structure of Malay BanLec beyond disruption of pi–pi stacking, as was the case with H84T versus WT BanLec. In order to compare F84T and WT Malay BanLec, we solved their crystal structures. First, we aligned the structure of WT Malay BanLec to that of WT BanLec (Fig. 6A). The structures are remarkably similar, with a root-mean-square deviation (RMSD) between all atoms of only 0.517 Å. Like BanLec, Malay BanLec has a β-prism-I fold characteristic of the jacalin-related lectin family. Both Malay BanLec and BanLec have a pi–pi stacking interaction between amino acid 84 (F and H, respectively) and Y83 within the ligand recognition group between CBS I and II. However, the aromatic rings involved in pi–pi stacking are arranged in a planar (sandwich) conformation in BanLec, whereas they are arranged in an edge-on conformation in Malay BanLec (Fig. 6B). The F84T mutation in Malay BanLec disrupts pi–pi stacking without significant changes to the overall structure of the monomer (Fig. 6C) or to the dimer (Fig. 6D). F84T and WT Malay BanLec have an almost identical structure, with an RMSD of 0.424 Å between all atoms.

## Discussion

Lectins have potential as antiviral agents as they are specific for their target glycans, but clinical development of promising broad-spectrum antiviral lectins has been halted for many of these molecules by the inability to separate mitogenic from antiviral activity^13^. The ability to fine-tune lectin function by separating distinct activities of lectins, such as mitogenicity and inhibition of viral replication, would be of great usefulness in designing therapeutic lectins with reduced mitogenicity (and perhaps increased efficacy), and in understanding the basic functions of lectins. We showed in our previous work with BanLec^15^, in the first apparent example of its kind, that the separation of mitogenic and antiviral activity is possible via targeted molecular engineering. A single amino acid substitution (H84T) that disrupts pi–pi stacking in the ligand recognition loop of the molecule is associated with the decrease in mitogenicity. Here we again demonstrate that disruption of pi–pi stacking in the ligand recognition loop of another lectin, Malay BanLec, is able to separate mitogenic and antiviral activity to a less dramatic but clear degree. It is quite exciting that molecular engineering aimed to eliminate the same structural element (pi–pi stacking) with the same substituted amino acid (threonine 84) resulted in separation of mitogenic and antiviral activities in two lectins, BanLec and Malay BanLec, that are structurally related but only ~ 60% identical at the amino acid level. This is to our knowledge only the second instance of separation of two distinct lectin functions, and confirms that, while pi–pi stacking is found at active sites in only a select number of lectins, molecular fine-tuning of lectins according to structural principles is feasible and can be predicted.

In our paper describing the molecular engineering of BanLec, multiple different substitutions were made for the histidine at position 84 and the resulting mutant proteins subjected to extensive NMR and molecular dynamic modeling, which, along with the crystallography, revealed that eliminating pi–pi stacking led to energetics favoring one, rather than two, binding pockets. This was due to the fall of the “mitogenic wall” that separates the two binding pockets^15^. These studies and glycocluster analysis led to the observation that H84T BanLec was much less active in multivalent interactions. As mitogenicity is thought to result from crosslinking of two or more molecules on a T cell (or perhaps an antigen-presenting cell), it appears that this is why H84T BanLec was far less mitogenic than is the parent lectin. F84T Malay BanLec similarly loses pi–pi stacking (the “mitogenic wall”), but otherwise retains a structure that is almost identical to its parent lectin. We thus conclude that F84T is less mitogenic than is WT Malay BanLec because it likely does not crosslink surface molecules as well. On the other hand, multivalent interactions are not expected to be needed for antiviral activity, as for this function the lectin simply must bind to the surface protein of the virus, and so H84T and F84T maintain their broad-spectrum antiviral activity.

F84T itself clearly still retains a high degree of mitogenic activity and is thus not a likely candidate for clinical development. However, we provide proof-of-concept that pi–pi stacking within the ligand recognition loop of a jacalin-related lectin is a determinant of mitogenicity that can be disrupted to reduce mitogenic activity without abolishing antiviral activity. Given the high mitogenic activity retained by F84T, it is likely that other elements confer mitogenicity in this lectin, though incomplete removal of endotoxin from F84T might also account for some mitogenicity. There are perhaps other determinants of mitogenicity to discover by comparing a still mitogenic lectin (F84T) to a non-mitogenic lectin (H84T), as Malay BanLec and BanLec differ at ~ 40% of their amino acid sequences. The H84T mutation is thought to disrupt the multivalent interactions required for mitogenic stimulation by decreasing binding of glycans to CBS II^15^; the mitogenicity of lectins depends on the ability to cross-link receptors, such as the T cell receptor, on immune cells^12^. It is therefore possible that the F84T mutation does not fully interfere with the ability of the lectin to participate in multivalent interactions such as those that would occur when cross-linking immune cell surface receptors, and so it retains some mitogenicity. Of particular interest to investigate would be the specific amino acid residues that differ within the carbohydrate binding sites and ligand recognition loops between F84T and H84T. Comparing antiviral lectins that are naturally mitogenic (such as Malay BanLec and BanLec) and those that are non-mitogenic (such as GRFT) could also help elucidate determinants of mitogenicity that could be removed without destroying antiviral activity.

On a related topic, it should be noted that precisely how lectins induce mitogenicity is not completely understood. As we reference above, cross-linking of T cell receptors (TCR) by lectins is proposed to mediate mitogenicity, at least for certain lectins^12,15^, but how the lectin and the TCRs interact is not known. It is presumed that they interact via binding to glycans on TCRs, as TCRs are glycosylated. Part of the difficulty in elucidating where and how lectins might bind to TCRs is that a full understanding of how lectins bind to glycans beyond mono- and di-saccharides has not yet been attained. While it is clear that the amino acid residues within the CBS and ligand recognition loops are important in determining binding specificity to different glycans^24^, we have not yet achieved the ability to precisely predict to which complex glycans a lectin with known specificity will or will not bind. For example, though BanLec is known to have high-mannose-binding activity, it does inhibit all viruses that express high-mannose *N*-glycans (e.g., respiratory syncytial virus), which may mean that it cannot efficiently bind to these viruses (unpublished data). The ability to decrease mitogenic activity in naturally mitogenic lectins is likely to lead to experiments that help illuminate which specific interactions with cells lead to mitogenicity.

In sharp contrast to many of the currently available antiviral therapies, lectins often have broad-spectrum antiviral activity, which would be beneficial for both targeted and empiric treatment of viral illnesses. Indeed, both H84T and F84T have broad-spectrum antiviral activity across a range of viruses from different families, including HIV, Ebola virus, and varicella-zoster virus, as they bind and presumably bind, respectively, to high-mannose type *N*-glycans that are on the surface of many viruses. Interestingly, the mechanism of action by which lectins inhibit viruses varies by the virus, with WT BanLec inhibiting HIV at the step of attachment^14^, and H84T inhibiting influenza virus at the step of fusion^16^ and Ebola virus at the step of entry as well as transcription/replication^17^. Though some lectins do appear to bind to certain cells^23^, the antiviral activities of lectins appear to be based on binding to the virus rather than to the cell, as lectins bind directly to the glycosylated membranes of viruses in a glycan-dependent manner; the specific glycans recognized by antiviral lectins, such as high-mannose, are generally not found on healthy human cells. For example, F84T (Fig. 4) and WT BanLec^14^ bind to HIV envelope protein and, in a high-mannose-specific manner, H84T binds to influenza HA but does not hemagglutinate red blood cells^16^, which suggests that H84T does not significantly interact with red blood cell surface glycans.

In this work, we demonstrate that Malay BanLec and BanLec are structurally highly similar, though they share only ~ 60% amino acid identity. Disruption of the pi–pi stacking interaction is achieved via the F84T mutation in Malay BanLec without overall structural changes to the lectin. As hypothesized, taking down this “mitogenic wall”^15^ leads to an overall decrease in mitogenicity. Importantly, F84T retains the ability to bind to HIV and retains antiviral activity against multiple strains of HIV-1 and HIV-2 with different tropisms. It is also broad-spectrum, being quite active against human cytomegalovirus, and varicella-zoster, Ebola, and Lassa fever viruses. In contradistinction to most healthy human cells, a number of malignancies display high-mannose or other unusual carbohydrates on their surfaces^25–27^, and thus molecularly engineered lectins also hold promise as anti-cancer agents. Lectins are specific for their targets, having evolved for millions of years, and have shown great antiviral potential that has been limited by the mitogenicity of many of these proteins. However, if we could harness a greater understanding of the mitogenic and effector elements of lectins, as this work provides evidence is possible, we might be able to unlock the antiviral and anti-cancer potential of lectins to treat an array of human illnesses.

## Methods

### Lectin production

Recombinant His-tagged D133G BanLec, WT Malay BanLec, and F84T Malay BanLec were produced in and harvested from *E. coli* as previously described^15^. Non-His-tagged H84T, which is used throughout this study, was produced similarly, except that Sephadex G-75 columns were used in place of Ni–NTA agarose columns for purification and elution was performed with 0.2 M methyl-α-D-glucopyranoside. Endotoxin testing for all lectins was performed with the Pierce LAL Chromogenic Endotoxin Quantitation Kit (Thermo Fisher Scientific). Endotoxin removal was then performed by adding 1 M glucose and passing pooled eluates containing protein through Mustang E filters (Pall). Endotoxin removal was performed to yield lectin with < 0.1 endotoxin units/mg of protein for lectins besides F84T and WT Malay BanLec; removal of endotoxin from these lectins was less efficient. After removal of endotoxin, glucose was removed and lectin concentrated in water using the Vivaspin 20 centrifugal unit with 3 K MWCO. Finally, endotoxin and protein concentrations were measured via LAL and BCA assays, respectively.

### F84T mutation of Malay BanLec

Site-directed mutagenesis to introduce the F84T mutation into Malay BanLec was performed using the QuikChange Lightning Site-Directed Mutagenesis Kit (Agilent) according to the manufacturer’s instructions. Sense and antisense primers were designed using the QuikChange Primer Design software: sense 5′-catggagggtcacgtggttgattatactggcctgaccatc-3′ and antisense 5′-gatggtcaggccagtataatcaaccacgtgaccctccatg-3′. Two nucleotide changes were introduced via these primers to change the F84 codon (TTT) to a threonine codon (ACT). Following the PCR reaction, plasmids were transformed into BL21-CodonPlus Competent Cells and colonies screened by Sanger sequencing for successful mutagenesis.

### Assessment of anti-HIV activity by TZM-bl cell infection

As previously described^15^, 100 μl of a 1 × 10^5^ cells/ml suspension of TZM-bl cells (HELA cells engineered to express CD4, CXCR4, and CCR5 and containing the HIV long terminal repeat upstream of a luciferase gene) were added to each well of a white 96-well plate. Cells were pre-treated with lectins (or PBS as a control) at 2X the final concentration for 30 min before the addition of 100 μl of the HIV strain BaL, with lectins maintained in the media throughout the experiment. Two days post-infection, medium was removed and 100 μl of ONE-Glo Luciferase reagent (Promega) added to determine luciferase expression.

### Isolation of PBLs and PBMCs

Peripheral blood mononuclear cells (PBMCs) were isolated from buffy coats from healthy volunteer donors (Red Cross Flanders, Leuven, Belgium) by density gradient centrifugation. Ethical clearance was provided by the Red Cross Flanders (file number M20190508A). Peripheral blood lymphocytes (PBLs) were isolated as above in a protocol approved by the University of Michigan Institutional Review Board. Informed consent was provided by all donors and all work was conducted in accordance with the guidelines and regulations of the bodies listed above.

### Assessment of mitogenic activity by BrdU incorporation in PBLs

As previously described^15^, PBLs were isolated and resuspended in RPMI medium containing 10% fetal bovine serum at a concentration of 2 × 10^6^ cells/mL. 50 μL of cell suspension were added per well of a white 96-well plate followed by 50 μL of medium containing lectins at various concentrations or PBS. Samples were deidentified prior to use in the study. The cells were incubated at 37 °C for 3 days prior to an 18 h addition of BrdU. Proliferation was measured by BrdU incorporation, which was detected via a chemiluminescence-based ELISA (Cell Proliferation ELISA (chemiluminescent); Roche) as per the manufacturer’s instructions.

### Evaluation of cellular activation markers

PBMCs treated for 3 days with the indicated concentrations of lectins were analyzed by flow cytometry following dual fluorescent staining with anti-mouse antibodies from BD Pharmingen (San Diego, CA), as has been described previously^23^. Samples were deidentified prior to use in the study. Briefly, cell cultures were transferred from plates to 5 ml round bottom tubes and washed with PBS containing 5% inactivated FBS (washing solution). After 10 min blocking with purified rat anti-mouse CD16/CD32 (Mouse BD Fc Block), cells were incubated in the dark with FITC-conjugated anti-CD4 mAb in combination with PE-conjugated anti-CD25 or anti-CD69 mAb for 30 min on ice. Finally, PBMCs were washed and analyzed with a FACSCelesta flow cytometer (BD, San Jose, CA), counting 10,000 events per sample. Data were acquired and analyzed using FlowJo from BD. PHA and GRFT were used as positive and negative controls, respectively.

### Bio-Plex cytokine/chemokine assay of human PBMC supernatants

PBMCs from multiple blood donors were cultured for 72 h in the presence of 10 µg/ml WT or F84T Malay BanLec, 10 µg/ml GRFT, or 2 µg/ml PHA. In the culture supernatants, the concentrations of IL-1α, IL-1ra, IL-2, IL-4, IL-5, IL-6, IL-7, IL-8, IL-9, IL-10, IL-12, IL-13, IL-15, IL-17, eotaxin, FGF, G-CSF, GM-CSF, IFN-γ, IP-10, MCP-1, MIP-1α, MIP-1β, PDGF-BB, RANTES, TNF-α, and VEGF were determined by the Bio-Plex 200 system (Bio-Rad) and Bio-Plex Human Cytokine 27-plex assay according to the manufacturer's instructions. Data were generated with the Bio-Plex Manager 4.1 software.

### Surface plasmon resonance (SPR) analysis

SPR technology was used to determine the binding of WT Malay BanLec, F84T Malay BanLec, and GRFT to immobilized gp140 on a Biacore T200 instrument (GE Healthcare, Uppsala, Sweden). BG505 SOSIP.664 gp140 trimers^28^ (kindly provided by Dr. R.W. Sanders) were immobilized on an NTA sensor chip using a capture coupling method. First, 0.5 mM Ni^2+^ was injected for 60 s to activate the NTA surface, then EDC/NHS 1:1 was injected for 60 s to activate the carboxyl groups. Following this, the gp140 trimers were captured and covalently bonded onto the surface by injecting them for 120 s with a concentration of 0.25 µg/ml diluted in HBS-P + (10 mM HEPES, 150 mM NaCl, 0.05% surfactant P20; pH 7.4) immobilization buffer. Afterwards, the surface was deactivated by a 60 s injection of 1.0 Methanolamine-HCl pH 8.5 and regenerated with 350 mM EDTA to remove any remaining bound ligand. All steps of the immobilization were performed with an injection speed of 10 µl/min. Interaction studies were performed at 25 °C in HBS-EP + (10 mM HEPES, 150 mM NaCl, 30 mM EDTA, 0.05% surfactant P20; pH 7.4). The lectins were serially diluted, covering a concentration range between 0.63 and 10 nM by using twofold dilution steps for WT and F84T Malay BanLec and between 0.12 and 10 nM using threefold dilution steps for GRFT. Samples were injected, using single cycle kinetics, for 2 min at a flow rate of 30 µl/min and the dissociation was followed for 10 min after the final injection. The sensor chip surface was regenerated with a 20 s injection of 50 mM NaOH followed by a 30 s injection of 10 mM glycine–HCl pH 1.5. For WT and F84T Malay BanLec, this regeneration step was performed twice as they were difficult to regenerate. Several buffer blanks were used for double referencing. Apparent binding kinetics (K_D_, k_a_, k_d_) were derived after fitting the experimental data to the 1:1 binding model in the Biacore T200 Evaluation Software 3.1.

### Anti-HIV cellular assays

We adapted the protocol as described in detail previously^29^. Briefly, CD4^+^CXCR4^+^ MT-4 cells (1 × 10^6^ cells/ml; 50 µl) were seeded in cell culture medium in a 96-well plate (Falcon, BD Biosciences, Erembodegem, Belgium) and pre-incubated with fivefold dilutions of test compound (100 µl) at 37 °C for 30 min, in duplicate. Virus (50 µl; HIV strains NL4.3, HE, or ROD) was added according to the 50% cell culture infectious dose (CCID_50_), which was determined by titration of the stock. After an incubation period of 5 days, virus-induced cytopathic effect (CPE) was scored with light microscopy and 50% inhibitory concentration (IC_50_) was calculated using the spectrophotometric MTS/PES viability staining assay (Cell-Titer96 Aqueous One Solution Proliferation Assay kit; Promega, Leiden, The Netherlands). Absorbance was measured using the Versamax microplate reader and SoftMax Pro software (Molecular Devices, Sunnyvale, CA, USA). To determine the potential cellular cytotoxicity of the compounds, the assays were also performed without the addition of virus. The reference compounds AMD3100 or maraviroc were included as controls in all assays.

The HIV-1 p24 Ag ELISA-based antiviral assay was performed in PBMCs, as described previously^30^. PHA-stimulated PBMCs (5 × 10^5^ cells/ml; 200 µl) were seeded in 48-well plates (Costar, Elscolab NC, Kruibeke, Belgium) and pre-incubated for 30 min with various concentrations of test compound (250 µl) in the presence of 2 ng/ml interleukin 2 (IL-2) (Roche Applied Science, Vilvoorde, Belgium). Next, virus (HIV-1 NL4.3, HE, or BaL) was added and 2 ng/ml IL-2 was supplemented again at day 3 and 6 after infection. After 10 days of infection, supernatant was collected and stored. Viral replication was determined by HIV-1 p24 Ag ELISA (Perkin Elmer) according to the manufacturer’s instructions.

### Anti-varicella-zoster virus (VZV) cellular assays

VZV plaque reduction assays were performed as described previously^31^. Briefly, human foreskin fibroblast (HFF) cells were seeded into 6-well plates. After seeding (48 h), they were infected with virus at a concentration that would yield 25–30 plaques per well. After 1 h incubation, the drug was serially diluted 1:5 at 6 different concentrations and added to the wells. The plates were incubated for 10 days, stained with a 1% neutral red solution (Sigma), and counted using a stereomicroscope. 50% effective concentration (EC_50_) and 50% cytotoxic concentration (CC_50_) values were calculated using the MacSynergy program.

### Anti-CMV cellular assays

CMV titer reduction assays were performed as follows: 96-well microtiter plates were seeded 24 h prior to infection. Cells were then infected with a high MOI of either AD169 or GDGrK17 virus for 1 h. The cells were washed and 7 concentrations of serially diluted drugs were added. These plates were incubated for 72 h. The plates were then freeze-thawed twice and spun to remove debris. Using a BioMek 4000 autodilutor, the supernatants were serially diluted into a 384-well plate containing HFF cells. After 14 days the plates were either stained with CellTiter Glo (Promega) and read on a luminometer or the DNA extracted with Extracta (Quanta Bio) and qPCR assays performed. 90% effective concentration (EC_90_) values were calculated using an Excel program.

### Cytotoxicity and plaque reduction assays for assessment of anti-Ebola and Lassa fever virus activity

The cytotoxicity assay (In vitro Toxicology Assay Kit, Neutral red based; Sigma) was performed in 96-well plates following the manufacturer’s instructions. Briefly, growth medium was removed from confluent Vero E6 cell monolayers and replaced with fresh medium (total of 100 µl) containing F84T. Control wells contained medium with the positive control or medium devoid of lectin. Wells without cells and growth medium only served as blanks. A total of up to five replicates were performed for each condition. Plates were incubated for 5 days (length of incubation time for Lassa virus plaque assay) and 10 days (length of incubation time for Ebola virus plaque assay), respectively, at 37 °C with 5% CO_2_. The plates were stained with 0.033% neutral red for approximately two hours at 37 °C in a 5% CO_2_ incubator. The neutral red medium was removed by complete aspiration, and the cells rinsed 1 × with PBS to remove residual dye. The PBS was completely removed and the incorporated neutral red eluted with 1% acetic acid/50% ethanol for at least 30 min. The dye content in each well was quantified using a 96-well spectrophotometer at 540 nm wavelength and 690 nm wavelength (background reading). The CC_50_ values were calculated by linear regression analysis.

For the plaque reduction assay, confluent or near-confluent Vero E6 cell culture monolayers in 12-well disposable cell culture plates were prepared. Cells were maintained in DMEM supplemented with 10% FBS. For antiviral assays the same medium was used but with FBS reduced to 5% or less and supplemented with 1% penicillin/streptomycin. F84T was prepared in 2 × DMEM and mixed 1:1 with virus prior to infection of cells. The virus control and cell control were run in parallel and the assay performed in biological triplicate. The assay was initiated by first removing growth media from the 12-well plates of cells, and infecting cells with 0.01 multiplicity of infection (MOI) of virus or about 50 to 100 plaque forming units (pfu) in the presence of lectin. Cells were incubated for 60 min with 100 μl inoculum/well, at 37 °C, 5% CO_2_ with constant gentle rocking. For Lassa virus, virus inoculum was removed and cells washed and overlaid with 0.8% tragacanth diluted 1:1 with 2 × DMEM and supplemented with 5% FBS, 1% penicillin/streptomycin, and the corresponding lectin concentration. For Ebola virus, virus inoculum was removed and cells washed and overlaid with 0.5% methylcellulose diluted 1:1 with 2 × DMEM and supplemented with 2% FBS, 1% penicillin/streptomycin, and the corresponding lectin concentration. Cells were incubated at 37 °C with 5% CO_2_ for 5 days for Lassa virus and for 10 days for Ebola virus. Plotting the log_10_ of the inhibitor concentration versus log_10_ of virus produced at each concentration allowed calculation of the EC_50_ by linear regression.

### Crystallization and structure determination of WT and F84T Malay BanLec

WT and F84T Malay BanLec were each concentrated to 5 mg/mL in buffer containing 10 mM HEPES, pH 7.5 and 150 mM NaCl. Crystals of WT Malay BanLec grew at 20 °C in drops containing equal volumes of protein and precipitating solution (1.6 M ammonium sulfate, 2% polyethylene glycol 2 K, and 0.1 M HEPES, pH 7.5). Prior to data collection, crystals were cryoprotected in well solution containing 20% ethylene glycol. Crystals of F84T Malay BanLec also grew at 20 °C from drops containing equal volumes of protein and well solution (1.8 M ammonium sulfate, 5% polyethylene glycol 400, and 0.1 M MES, pH 6.5) and were cryoprotected in 1.8 M lithium sulfate, 5% polyethylene glycol 400, and 0.1 M MES, pH 6.5. Data were collected at the Life Sciences Collaborative Access Team beamline 21-ID-G at the Advance Photon Source at Argonne National Laboratory. Data were processed with HKL2000^32^ and the structures were solved by molecular replacement using Phaser^33^ for WT and MolRep^34^ for F84T Malay BanLec with PDB 2BMY as a search model. The structures were iteratively refined with Buster^35^ and fit with COOT^36^. WT Malay BanLec crystallized in the P4_3_2_1_2 space group with 1 dimer in the asymmetric unit and diffracted to 1.5 Å resolution. F84T Malay BanLec crystallized in space group P2_1_2_1_2_1_ with 8 dimers in the asymmetric unit and diffracted to 1.8 Å resolution. See Table S1 for data collection and refinement statistics.

## Supplementary Information

Supplementary Information

## Data Availability

H84T and F84T must be obtained via MTA. Structures of WT and F84T Malay BanLec were deposited in the Research Collaboratory for Structural Bioinformatics Protein Data Bank (PDB). The PDB ID for WT Malay BanLec is 7KMU; that for F84T is 7KMV.
