# Supplementary material for: Targeted disruption of pi–pi stacking in Malaysian banana lectin reduces mitogenicity while preserving antiviral activity
Source: Sci Rep. 2021 Jan 12;11:656. doi: 10.1038/s41598-020-80577-7 (PMC7804308; doi:10.1038/s41598-020-80577-7)
Supplement: Supplementary file 1 — Supplementary Information [file 41598_2020_80577_MOESM1_ESM.pdf]

Supplementary Information

Targeted disruption of pi-pi stacking in Malaysian banana lectin reduces mitogenicity while preserving antiviral activity

Evelyn M. Covés-Datson[1,2], Steven R. King[3], Maureen Legendre[3], Michael D. Swanson[4,12], Auroni Gupta[3], Sandra Claes[5], Jennifer L. Meagher[6], Arnaud Boonen[5], Lihong Zhang[7], Birte Kalveram[7], Zoe Raglow[3], Alexander N. Freiberg[7], Mark Prichard[8], Jeanne A. Stuckey[6,9], Dominique Schols[5], David M. Markovitz[3,4,10,11]*

[1]Medical Scientist Training Program, University of Michigan, Ann Arbor MI 48109; [2]Department of Microbiology & Immunology, University of Michigan, Ann Arbor MI 48109; [3]Department of Internal Medicine, Division of Infectious Diseases, University of Michigan, Ann Arbor MI 48109; [4]Graduate Program in Immunology, University of Michigan, Ann Arbor MI 48109; [5]Laboratory of Virology and Chemotherapy, Rega Institute for Medical Research, University of Leuven, 3000 Leuven, Belgium; [6]Life Sciences Institute, University of Michigan, Ann Arbor MI 48109; [7]Department of Pathology, University of Texas Medical Branch, Galveston TX 77555; [8]University of Alabama Health Services Foundation Diagnostic Virology Laboratory, University of Alabama at Birmingham, Birmingham AL 35294; [9]Department of Biological Chemistry, University of Michigan, Ann Arbor MI 48109; [10]Cellular and Molecular Biology Program, University of Michigan, Ann Arbor MI 48109; [11]Cancer Biology Program, University of Michigan, Ann Arbor MI 48109; Present Address: Predictive and Clinical Immunogenicity, Merck and Co., Inc, Kenilworth NJ 07033

Material included:

Table S1

Table S1. Crystallography Data Collection and Refinement Statistics.

**Data Collection**	**WT Malay BanLec**	**F84T Malay BanLec**
PDBID	6OPR	6OQ0
Space Group	$P4_32_12$	$P2_12_12_1$
Unit Cell (Å)	52.526, 52.526, 221.307	79.247, 157.871, 204.101
Wavelength (Å)	0.9786	0.9786
Resolution (Å)[1]	1.51 (1.51 – 1.54)	1.80 (1.80 – 1.83)
Rsym[2]	0.060 (0.414)	0.084 (0.662)
<I/σI>[3]	20 (5)	10 (2)
Completeness (%)[4]	99.5 (98.4)	99.8 (99.5)
Redundancy	14.0 (11.7)	7.1 (6.9)
**Refinement**		
Resolution (Å)	1.51	1.80
R-Factor[5]	18.4	20.2
Rfree[6]	20.2	23.4
Protein atoms	2117	16861
Water Molecules	363	2240
Unique Reflections	49784	229852
R.m.s.d.[7]		
Bonds	0.008	0.008
Angles	0.99	1.07
MolProbity Score (1)	0.92	0.93
Clash Score (1)	0.70	0.76

[1]Statistics for highest resolution bin of reflections in parentheses.

[2]$R_{sym} = \Sigma_h \Sigma_j | I_{hj} - \langle I_h \rangle | / \Sigma_h \Sigma_j I_{hj}$, where I_{hj} is the intensity of observation j of reflection h and $\langle I_h \rangle$ is the mean intensity for multiply recorded reflections.

[3]Intensity signal-to-noise ratio.

[4]Completeness of the unique diffraction data.

[5]R-factor = $\Sigma_h | |F_o| - |F_c| | / \Sigma_h |F_o|$, where F_o and F_c are the observed and calculated structure factor amplitudes for reflection h.

[6]R_{free} is calculated against a 5% random sampling of the reflections that were removed before structure refinement.

[7]Root mean square deviation of bond lengths and bond angles.

References

1. Chen, V. B., Arendall, W. B., Headd, J. J., Keedy, D. A., Immormino, R. M., Kapral, G. J., Murray, L. W., Richardson, J. S., and Richardson, D. C. (2010) *MolProbity* : all-atom structure validation for macromolecular crystallography. *Acta Crystallogr. Sect. D Biol. Crystallogr.* **66**, 12–21
